# Evidence for human diabetic cardiomyopathy

**DOI:** 10.1007/s00592-021-01705-x

**Published:** 2021-03-31

**Authors:** Raffaele Marfella, Celestino Sardu, Gelsomina Mansueto, Claudio Napoli, Giuseppe Paolisso

**Affiliations:** Department of Advanced Medical and Surgical Sciences, University of Campania “Luigi Vanvitelli”, Piazza Miraglia 2, 80131 Naples, Italy

**Keywords:** Diabetic cardiomyopathy, Heart failure, Diabetes

## Abstract

Growing interest has been accumulated in the definition of worsening effects of diabetes in the cardiovascular system. This is associated with epidemiological data regarding the high incidence of heart failure (HF) in diabetic patients. To investigate the detrimental effects both of hyperglycemia and insulin resistance, a lot of preclinical models were developed. However, the evidence of pathogenic and histological alterations of the so-called diabetic cardiomyopathy (DCM) is still poorly understood in humans. Here, we provide a stringent literature analysis to investigate unique data regarding human DCM. This approach established that lipotoxic-related events might play a central role in the initiation and progression of human DCM. The major limitation in the acquisition of human data is due to the fact of heart specimen availability. Postmortem analysis revealed the end stage of the disease; thus, we need to gain knowledge on the pathogenic events from the early stages until cardiac fibrosis underlying the end-stage HF.

## Introduction

Cardiovascular disease (CVD) is the leading cause of mortality in patients with type 2 diabetes (T2D), [1]. Notably, CVD is directly linked to coronary atherosclerosis [1]. To date, dyslipidemia and hypertension are risk factors mainly involved in this disease, while it is not well known if diabetes could alone evoke molecular changes in the heart structure and function [2]. Recently, authors provided that diabetic cardiomyopathy (DCM) is an existing form of heart disease, which shows a distinctive remodeling pattern compared to other cardiomyopathies [3]. This concept was introduced by Rubler et al. in 1972, [4]. Indeed, these authors firstly evidenced DCM, as pathological condition of heart dysfunction in patients with healthy coronary arteries [4]. This condition was confirmed in 1974 by the Framingham Heart Study [5]. This study evidenced a higher rate of heart failure (HF) in diabetic women (fivefold) and men (2.4-fold) without association with other risk factors, such as age, coronary heart disease (CHD), and hypertension [5]. According to this study [5], more recently the American College of Cardiology Foundation [6] and the European Society of Cardiology, in collaboration with the European Association for the Study of Diabetes [7], described diabetic cardiomyopathy as a clinical condition of heart dysfunction without coronary atherosclerotic lesions and hypertension in diabetic patients. On other hand, authors defined the cardiomiopathy any condition of heart muscle disease with structurally and functionally abnormal myocardium in the absence of CHD and hypertensive, valvular, or congenital heart disorders [8]. In this context, current researches are focused on the detailed identification of the pathophysiological mechanisms leading to DCM in humans. Moreover, here we discuss the current knowledge of molecular and pathophysiological altered pathways causing DCM in humans.

## DCM clinical evaluation

DCM shows symptoms and signs comparable to a dilated cardiomyopathy caused by toxic agents or viral myocarditis [9]. Indeed, at clinical level the patients with DCM have symptoms and signs not different from those experienced by patients with heart failure with reduced ejection fraction (HFREF). On other hand, recently authors reported DCM as a heart disease different from other clinical manifestation of other forms of dilated cardiomyopathy [10]. Indeed, the patients with DCM are typically affected by obesity, T2D, and with a heart dysfunction caused by small left ventricular (LV) cavity, normal LV ejection fraction, thick LV walls, elevated LV filling pressures, and enlargement of left atrium [11]. By the way, these are the diagnostic findings more common seen in restrictive cardiomyopathy, and not diagnosed in patients with dilated cardiomyopathy. Indeed, the currently used definition of DMC is that based on evidence of a heart dysfunction with a restrictive phenotype [12]. Notably, this is due by the evidence of a normal-sized left ventricle with normal left ventricular ejection fraction (LVEF) in affected patients [12]. To date, these patients could be classified as patients affected by heart failure with preserved left ventricular ejection fraction (HFPEF), [13]. Furthermore, DMC patients could be diagnosed as patients with HFPEF [13]. Furthermore, this concept has been previously documented by authors, indicating T2D-related cardiomyopathy as a heart disease with restrictive/HFPEF phenotype [11, 14]. In addition, this phenotype could be diagnosed as a precursor stage of DCM with dilated/HFREF phenotype [11, 14]. These observations fit with the epidemiological data suggesting that individuals with T2D are at a high risk of developing congestive heart failure (HF), having a relative risk at least twice as high as individuals without diabetes, mainly when urinary albumin excretion rate (u-AER) is elevated [15]. HF is a severe complication in patients with T2D, with a median survival from diagnosis of 3.5 years [16] and a 5-year mortality rate of 75% [17]. Finally, the prognosis of patients with T2D and HF is worse than that of HF patients without diabetes [18].

## DCM instrumental evaluations

The aging could cause the cardiac remodeling by decreasing LV dimensions and increasing fractional shortening. T2D could diminish but it does not reverse this LV remodeling pattern [9]. Furthermore, we could say that LV dilatation could cause the shifting from asymptomatic stage to diagnosed form of HFPEF [19]. Thus, in the early stages, diabetic cardiomyopathy is characterized by a subclinical period with structural and functional anomalies, including left ventricular (LV) hypertrophy and subsequently reduced ejection fraction [20]. However, from the literature the T2D could be the leading cause of structural and functional cardiac alterations [21].

### Structural changes

The T2D patients as compared to patients without diabetes have higher LV mass, wall thickness, arterial stiffness, and reduced LVEF [22]. These alterations were independently from body mass index and blood pressure values [22]. In this setting, the Framingham Heart Study indicated that LV mass and wall thickness were principally altered among female patients with T2D [23]. Notably, these factors linked to the degree of glucose intolerance and obesity [23]. Thus, another pattern characteristic in T2D patients could be the evidence of concentric remodeling or increased regional wall thickness without left ventricular hypertrophy [24]. We know that this could have different effects on systolic or diastolic heart function [24]. On other hand, T2D-associated heart concentric hypertrophy, which is diagnosed by abnormal LV mass and wall thickness, could cause diastolic dysfunction [22]. Intriguingly, its worsening could lead to eccentric hypertrophy with systolic dysfunction [22].

### Functional changes

The initial stage of LV diastolic dysfunction in T2D patients could be evidenced by cardiac catheterization [25]. Indeed, normotensive T2D patients showed failing heart symptoms in absence of CHD [25]. Notably, these patients had increased LV end-diastolic pressure, decreased LV end-diastolic volume and a normal ejection fraction (EF). In this context, echocardiography with Doppler could be used to evaluate the diastolic herat function in a non-invasive way [13]. Indeed, the trans-mitral inflow, the flow velocities, the flow patterns, the isovolumic relaxation time, and the deceleration time are well-known indexes of diastolic heart function [13].

Intriguingly, in T2D patients we could have the reduction of LV ejection time, with increased pre-ejection period and ratio of the pre-ejection period to LV ejection time [13]. Therefore, the diastolic abnormalities could evidence the earliest functional alterations in patients with DCM [13]. On other hand, authors observed that systolic function parameters were normal in 20 patients with type 1 diabetes and in 20 patients with T2D [26]. However, they said that diastolic function was worsened in diabetic patients without evident CVD than in 12 healthy controls [26]. In addition, authors found that in normotensive asymptomatic T2D patients with good glycemic control the 47% had diastolic dysfunction [27]. This result increased up to the 75% of T2D patients regards the diastolic dysfunction abnormalities [28]. Moreover, noninvasive cardiac performance evaluation in T2D patients without evident heart dysfunction has demonstrated a prolonged pre-ejection performance and a shortened ejection period, both of which correlate with reduced resting LV ejection fraction (LVEF) and diminished systolic function [29]. Notably, T2D patients showed a lower LVEF in response to exercise, suggesting a reduction in cardiac reserve [30]. Thus, patients with T2D could evidence an early LV systolic dysfunction with normal LVEF [30]. This finding has been deeply investigated by more accurate systolic assessment techniques, such as strain, strain rate, and myocardial tissue Doppler velocity [28]. Indeed, this noninvasive cardiac echographic techniques might find preclinical systolic abnormalities in T2D patients with diastolic dysfunction [28].

## Progression of pathogenic events in humans

**Fig. 1:**
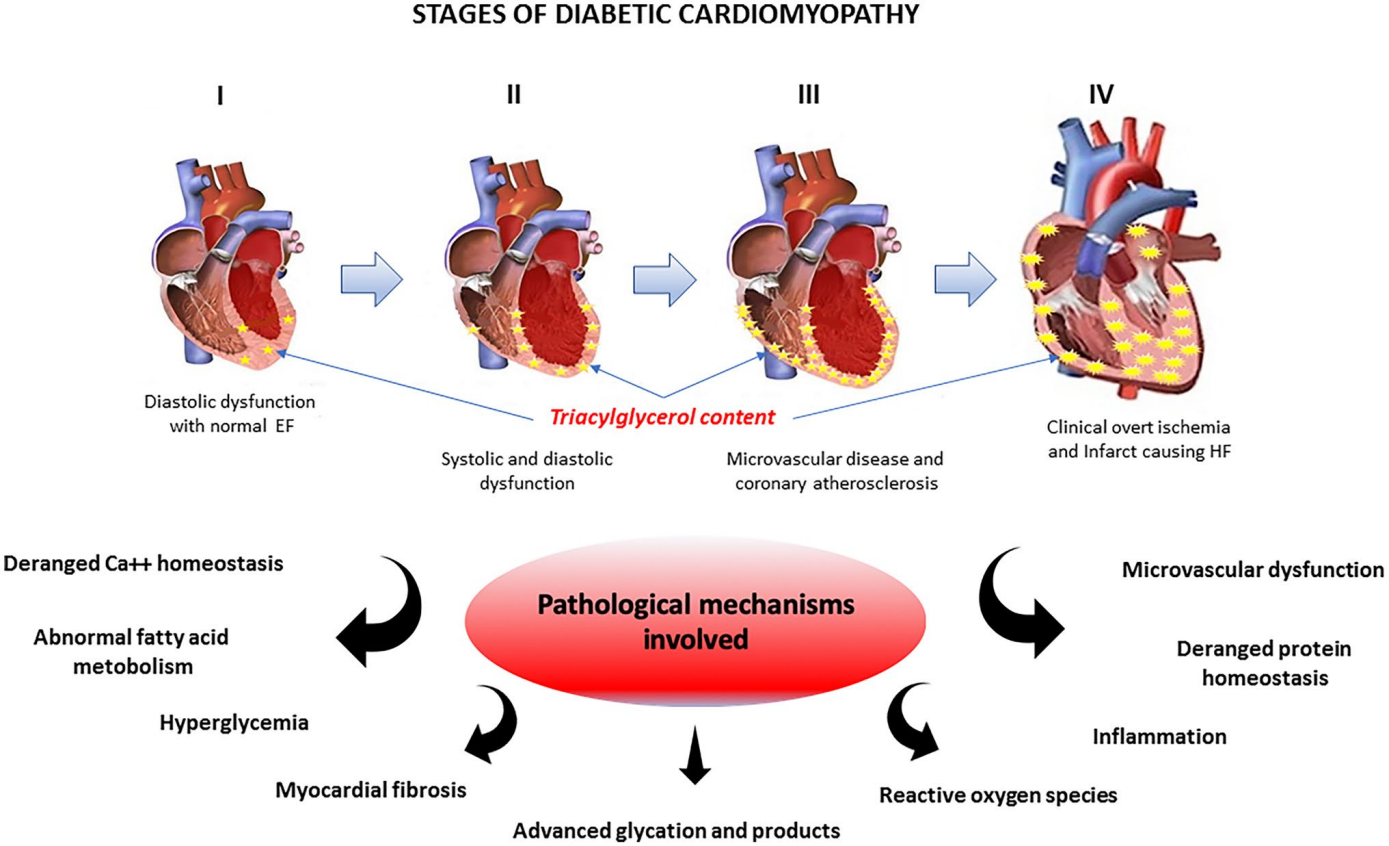
Role of myocyte lipid accumulation in diabetic cardiomyopathy progression

Many hypotheses have been formulated about the mechanisms underlying the etiology of DCM, as insulin resistance and hyperglycemia, with oxidative stress, inflammatory components, inappropriate modulation of immune properties, alteration of subcellular components, endothelial and coronary microcirculation abnormalities with apoptosis, fibrosis, inflammation, and lipid accumulation [3, 31]. These shreds of evidence of diabetic heart derived mainly from non-human experimental models. Besides, the data from these experimental studies are often conflicting and difficult to prove in humans. Therefore, we analyzed the current knowledge about the mechanisms implicated in the beginning and progression of human diabetic cardiomyopathy. Three different stages describe the evolution of diabetic cardiomyopathy with different pathophysiological characteristics and clinical outcomes (Fig. 1).

### Early stage

In this stage, we do not have significant changes in myocardial structure and systolic function by echocardiographic evaluations [32, 33] in the early stage of DCM. However, worsened myocardial relaxation can be found by echocardiography and MRI [34, 35]. However, the insulin resistance is a main trait of T2D, and whole-body insulin resistance is another aspect of left ventricle dysfunction (LVD) and congestive HF [36, 37]. Again, the severity of insulin resistance independently predicts mortality [38, 39]. Notably, the pathophysiology of organ-specific insulin resistance was poorly described in humans, but insulin-resistant syndromes influence cardiovascular morbidity and mortality in these patients [40]. Indeed, in insulin-resistant patients the reduced whole-body glucose disposal exhibits abnormalities of the skeletal muscle [40]. Thus, the initial deficit underlying myocardial insulin resistance in the diabetic heart appears at the level of insulin receptor substrate (IRS)-1/2 and glucose transport (GLUT4) vesicle trafficking and docking diminished insulin-stimulated activity [41]. Notably, the insulin resistance and hyperglycemia are associated with elevated plasma levels of non-esterified fatty acids (NEFAs) [42] and ectopic accumulation of triglycerides (TG), such as reflected in cardiac steatosis [43]. Furthermore, myocardial lipid accumulation was observed in the human heart with preserved ejection fraction from patients with insulin resistance and hyperglycemia [44]. However, in patients with metabolic syndrome there was a strong correlation between the progression of cardiac dysfunction and myocytes lipid accumulation because, with increase of vacuolated myocytes and oil red O staining-positive myocytes, myocardial performance index increases and EF decreases [44]. In this context, patients with metabolic syndrome showed increased protein levels of SREBP-1c and PPAR*γ*, and lower levels of SERCA2a [44]. However, this could suggest the stimulation of lipogenesis and the impaired calcium handling in this class of patients [44]. Intriguingly, authors found an association between these abnormalities and the degree of left ventricular dysfunction [44]. Furthermore, this could suggest an existing link between molecular pathways of lipogenesis and abnormal calcium handling with ventricular dysfunction [44]. Remarkably, these authors did not evidence differences in both total cholesterol and LDL cholesterol levels in patients with the presence of increased levels of triglycerides and reduced HDL cholesterol [44]. Parallelly, in patients with metabolic syndrome the degree of insulin resistance is associated with the amount of lipid in the human myocardium [44]. Therefore, the current data of the molecular mechanisms that affect the onset of cardiac dysfunction in diabetic patients may be identified with heart insulin resistance and myocardial lipid accumulation, as evidenced by imaging studies [45]. Finally, many epigenetic-sensitive mechanisms are active during diabetes [46, 47]. These epigenetic paths are both in circulating CD04/CD08 cells [48, 49] and in cardiovascular tissues [50], and they may be implicated in the pathogenesis of human DCM.

### Advanced stage

In patients with advanced stage of diabetic cardiomyopathy, the echocardiography could evidence several modifications of diastolic function (initially) and systolic function (later in the process), which are the result of increased cardiac fibrosis [26]. Thus, the heart biopsies could evidence interstitial fibrosis, myocyte hypertrophy, and contractile protein glycosylation increase in diabetics [51]. To date, all these pathological processes could cause reduced diastolic compliance and ventricular hypertrophy in diabetic patients [51]. In this setting, recently authors reported that accumulation of advanced glycation end products (AGEs) and collagen deposition are important features of the increased LV stiffness in patients with HF and reduced EF [30]. On other hand, a high cardiomyocyte resting tension could be the main determinant of increased LV stiffness in patients with heart failure and normal EF. In diabetic cardiomyopathy, increased fibrosis increased perivascular and intermyofibrillar fibrosis has been observed in human myocardial samples in the absence of CHD and hypertension [20, 21]. Therefore, the progression of myocardial fibrosis in diabetic cardiomyopathy is caused by the accumulation of stiff collagen and its crosslinking, cardiac interstitial fibrosis [20, 21]. To date, this could cause the gradual abolition of muscular fibrils with perivascular fibrosis, thickened and sclerotic small coronary vessels, and basement membrane thickening, as well as coronary microvascular sclerosis and micro-aneurysms [20, 21]. Nevertheless, to date, we do not know whether these alterations are effectively related to the diastolic alterations highlighted by the echocardiographic evaluation in humans.

### Late stage

Changes of the metabolism with abnormal neurohumoral activation, and development of myocardial fibrosis could promote the coronary microcirculation, then leading to diastolic function and systolic dysfunction in the late stage of the human DCM [31, 52, 53]. However, the altered myocardial insulin signaling could be responsible for reduced activation of endothelial nitric oxide synthase and reduced bioavailable nitric oxide levels [31, 52, 53]. Moreover, increased oxidative stress reduces nitric oxide, which increases the destruction of this molecule [31, 52, 53]. Increases in ROS and inflammation and decreases in bioavailable nitric oxide raise interstitial collagen deposition and crosslinking, associated with interstitial fibrosis and impaired myocardial relaxation [31, 52, 53]. Then, the profibrotic TGF-*β*1–SMAD signaling pathway increases myocardial collagen and fibronectin content and interstitial fibrosis in concert with impaired insulin signaling [54–58]. Moreover, in the late stages of human diabetic cardiomyopathy authors reported a negative clinical impact caused by tight cross-talking between inflammatory signals (i.e., A disintegrin and A metalloproteinase 1/TNF*α* signaling) and fibrosis [54–58]. Indeed, the alterations of DCM include cardiomyocyte necrosis with muscular fibril reduction, and increase formation and deposition of collagen in connective tissue [54–58]. However, this could than result in interstitial or/and perivascular fibrosis, cellular hypertrophy, thickened and sclerotic small coronary vessels, basement membrane thickening, hyaline arteriolar sclerosis, and capillary micro-aneurysms [54–58].

## Conclusions

Much advance is now gained in the pathogenesis of human DCM. However, we need to establish the precise chain of events from the early accumulation of lipotoxicity within the myocardium until the stage of advanced HF. A novel study model proposed in the transplanted heart has been proposed by our group. Long-term studies should be carried in this model, and the search for liquid biopsy biomarkers can be integrated into the light of precision medicine of human DCM.
